# Morphology of the sternoclavicular joint and its microanatomical changes in response to osteoarthritic degeneration

**DOI:** 10.1002/ca.24253

**Published:** 2024-12-20

**Authors:** Sophie Mok, Yousef Almajed, Abdulaziz Alomiery, Roger Soames, Abduelmenem Alashkham

**Affiliations:** ^1^ Anatomy, Biomedical Sciences University of Edinburgh Edinburgh UK; ^2^ Centre for Anatomy and Human Identification, School of Science and Engineering University of Dundee Dundee UK; ^3^ Zawia Faculty of Medicine University of Zawia Zawia Libya

**Keywords:** intra‐articular disc, sternoclavicular joint, sternoclavicular joint anatomy, sternoclavicular joint morphology, sternoclavicular joint osteoarthritis

## Abstract

Although the sternoclavicular joint shares structural similarities with the knee and hip joints as a diarthrodial joint, its biomechanics differ significantly due to its non‐weight‐bearing nature. Nevertheless, it is subject to considerable loading, leading to increased susceptibility to osteoarthritis, a prevalent condition characterized by the degeneration of the joint's articular surfaces and fibrocartilaginous intra‐articular disc. The osteoarthritic degeneration of the fibrocartilaginous and cartilaginous surfaces of the sternoclavicular joint has been investigated, considering multiple factors. These include cell count, collagen alignment, surface fibrillation, cyst formation, and glycosaminoglycan content, with the findings deemed significant. However, current treatments for osteoarthritis of the sternoclavicular joint tend to focus on symptom management rather than active prevention of disease progression. Therefore, a detailed understanding of the anatomy, biomechanics, and morphological changes of the sternoclavicular joint during all stages of the osteoarthritic disease is essential for effective management to allow for maximum patient outcomes. This review explores the current literature on the anatomy of the sternoclavicular joint, starting with its structure and comparison to surrounding joints, biomechanics, and morphology, before considering the microanatomical changes that occur due to osteoarthritic degeneration. Early identification of osteoarthritic changes within this joint can enhance treatment and management outcomes before advancing joint degeneration, improving the quality of life for those affected.

## 1 INTRODUCTION

The sternoclavicular joint (SCJ) forms the articulation between the clavicle, manubrium sterni and the 1st costal cartilage (Adams & Wigley, 2016). It plays a crucial role in orchestrating the movements of the shoulder girdle, demonstrating a range of three degrees of freedom, which include elevation, depression, protraction, retraction and axial rotation (Epperson & Varacallo, 2021; Palastanga & Soames, 2018a). Between the articular surfaces of the SCJ is an intra‐articular fibrocartilaginous disc, which protects and facilitates multidimensional movements at the joint (DePalma, 1959; Takeshige et al., 1998; Terry & Chopp, 2000). This disc is similar in function and composition to the intra‐articular disc present within the neighboring acromioclavicular joint (ACJ) of the shoulder girdle (Ghasemi et al., 2007; Takeshige et al., 1998; Terry & Chopp, 2000).

Given its integral role in governing all movements of the arm and shoulder, the SCJ forms one of the most frequently used joints in the body (Ghasemi et al., 2007). Consequently, it is susceptible to osteoarthritic degeneration. Similar to the general predisposing factors for osteoarthritis (OA) in other joints throughout the body, several factors increase the likelihood of OA development at the SCJ. These factors encompass prior injury, the extent of mechanical stress, advancing age, joint instability, and the presence of incomplete fibrocartilaginous discs (which can also emerge as a consequence of osteoarthritis) (Lawrence & East, 2017; Palazzo et al., 2016). Due to the involvement of the SCJ in all movements of the shoulder girdle, injury, or degeneration of this joint has a profound impact on the daily lives of affected individuals (Logan et al., 2018). Simple everyday tasks which include rotation, abduction and elevation of the arm at the shoulder may be impeded and result in pain as a result of SCJ degeneration or injury (Logan et al., 2018). In response to injury, rehabilitation protocols are in place, which may include immobilization and bracing, pain management, ice and rest (Bontempo & Mazzocca, 2010). However, a gold standard rehabilitation protocol has yet to be agreed upon across the literature (Logan et al., 2018). Surgical management of the injured SCJ typically involves treating joint instability, where a hamstring autograft is most commonly used to mimic external stabilization of the joint (Logan et al., 2018). Joint fixation has also been recommended through the use of a ‘locking plate’ and/or anchors (Escobar et al., 2022). Due to the joint proximity to neighboring neurovascular structures, fixation by pins is typically contraindicated (Escobar et al., 2022). Regardless of treatment methods, once injury of the SCJ has occurred, susceptibility to osteoarthritic degeneration increases substantially (Kiel et al., 2022).

**FIGURE 1:**
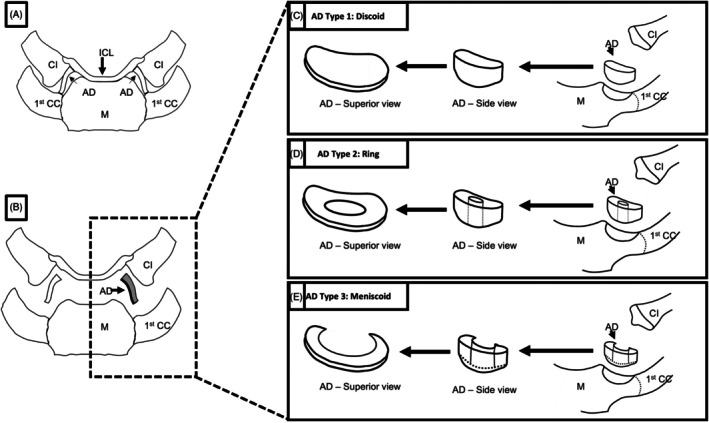
(A) Anterior view of the left and right sternoclavicular joints. The articular disc (AD) fully separates the joint cavity into two separate compartments. (B) A schematic illustration of the left sternoclavicular joint and AD. The AD of the sternoclavicular joint can be classified into three types: (C) type 1: Discoid, (D) type 2: Ring and (E) type 3: Meniscoid. The three AD types can be viewed in situ as in images A and B, as well as individually in images C, D and E. Cl: Clavicle, ICL: Interclavicular ligament, 1st CC: 1st Costal cartilage, M: Manubrium (DePalma, 1959; Palastanga and Soames, 2018a; Epperson & Varacallo, 2021).

It has been observed that OA leads to significant microanatomical changes in the fibrocartilaginous disc and hyaline articular cartilage of the clavicle and sternum (Emura et al., 2007; Ghasemi et al., 2007). Despite these observations, a grading system for evaluating changes in the fibrocartilaginous disc has yet to be established, leaving the relationship between fibrocartilage and articular cartilage degeneration of the SCJ unknown. The intra‐articular disc has been found to exhibit differences in shape, with some being discoid, annular (i.e., ring), or meniscal in morphology. While the intra‐articular disc of the ACJ has been described as meniscus‐like, it is uncertain whether these morphological variations are a result of osteoarthritis‐related degeneration or simply natural changes that occur over time (Figure 1) (de Palma, 1951; Emura et al., 2009; van Tongel et al., 2012).

Unlike the SCJ, the occurrence of incomplete intra‐articular discs of the ACJ, which are discs that have a free edge resembling the ‘C‐shape’ of a meniscus or a hole at the center, has been confirmed to be a result of age‐related degeneration. This degeneration of the disc, as reported by a study examining 168 ACJ's from 85 cadavers, typically precedes the deterioration of the surrounding joint cartilage (Petersson, 1983). Highlighting this finding further is a series of studies surrounding similar diarthrodial joints (knee and hip), which suggest that degeneration of fibrocartilaginous structures precedes osteoarthritic destruction in articular cartilage and bone (Domzalski et al., 2017; Dyment et al., 2015; Pauli et al., 2011). Although the biomechanics of the SCJ diverge from those of the knee and hip, primarily as it is not classified as a weight‐bearing joint, it has been established to undergo substantial loading (Bergmann et al., 2007). This loading is attributed to pronounced glenohumeral contact forces generated during routine daily activities (Klemt et al., 2018). Therefore, the osteoarthritic condition of the SCJ could potentially follow a comparable disease progression pattern, with initial degeneration beginning in the fibrocartilaginous intra‐articular disc prior to cartilaginous involvement. Examination of the microanatomical changes in fibrocartilaginous structures within a joint may therefore act as an early indicator of osteoarthritic degeneration, prior to the initiation of articular cartilage involvement and subsequent onset of pain and inflammation (Dyment et al., 2015).

Identification of the relationship between microanatomical changes within the intra‐articular disc, the cartilaginous surfaces of osteoarthritic SCJ and disc types will enhance the knowledge of osteoarthritic disease progression, as well as enable the diagnosis of early‐stage osteoarthritic degeneration. This review evaluates current findings on the microanatomical structure and function of normal and pathological SCJs in an attempt to provide a cohesive understanding of the role of osteoarthritis. It also highlights the lack of research surrounding the osteoarthritic SCJ, with a particular focus on the relationship between microanatomical changes of the intra‐articular disc and the articular cartilage.

## 2 ANATOMICAL STRUCTURE OF SCJ

**FIGURE 2:**
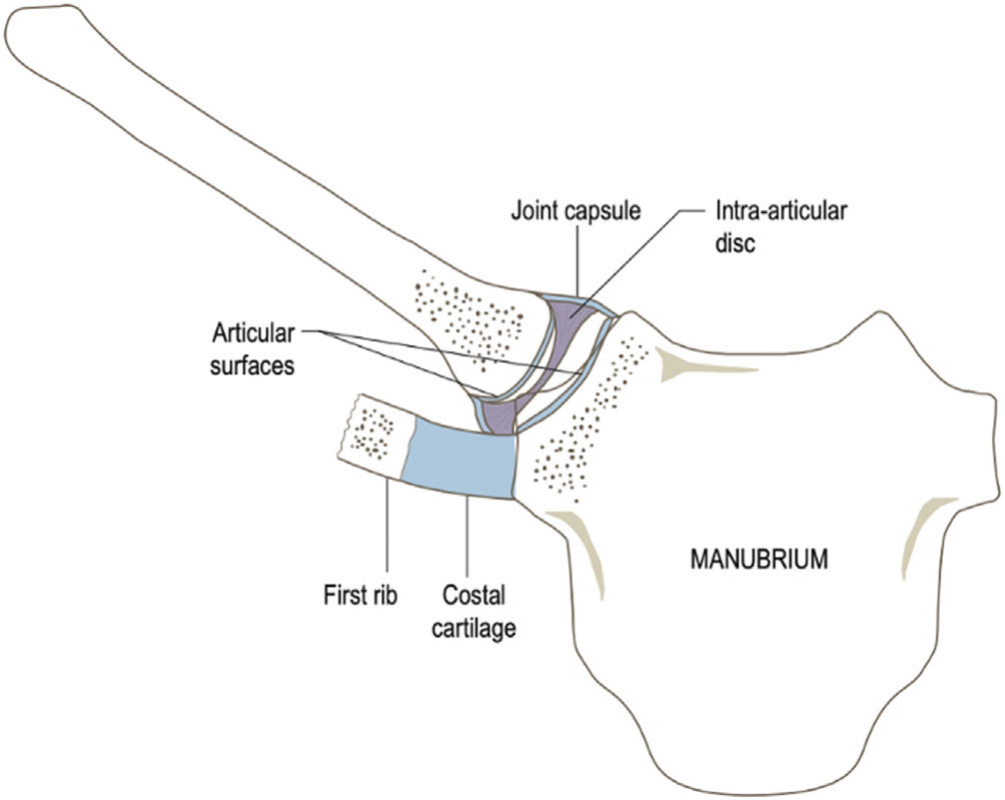
Anterior view of right Sternoclavicular joint and right clavicle. The medial end of the clavicle articulates with the manubrium sterni and 1st costal cartilage to form the sternoclavicular joint. An intra‐articular disc separates the joint cavity into two compartments; disc originates from 1st costal cartilage as a continuation of the intra‐articular ligament and inserts superoposteriorly into the articular cartilage of the clavicle and joint capsule (Palastange & Soames, 2012).

The SCJ provides the only true synovial connection between the trunk and upper extremity, being one of the four joints involved in the shoulder girdle complex (Epperson & Varacallo, 2021). It comprises articulations between the sternal end of the clavicle, the clavicular notch of the manubrium sterni and 1st costal cartilage (Figures 1 and 2) (van Tongel et al., 2012). The articular surface of the medial clavicle is typically larger than the articulating surface of the sternum and therefore the joint surfaces are not particularly congruent (Palastanga & Soames, 2018a). This mismatch in articulation between the medial clavicle and sternum results in poor osseous stability (Palastanga & Soames, 2018a). The SCJ is therefore contingent on the support provided by its intrinsic fibrocartilaginous disc and adjacent ligamentous structures (Olivier et al., 2022). The surrounding sternoclavicular (anterior and posterior), interclavicular and costoclavicular ligaments collectively form the primary stabilizers of this joint (Sewell et al., 2013). The synergistic action of these intrinsic and extrinsic ligaments collectively contributes to robust stability within the SCJ.

Across the literature, the SCJ has interchangeably been described as either a ‘diarthrodial joint’ or a ‘unique diarthrodial joint’. Typical diarthrodial joints are composed of articulating surfaces lined with articular cartilage, surrounded by a synovial membrane lined joint capsule (Mow et al., 1992). The joint surfaces of the SCJ have been described to be lined with articular cartilage as well as fibrocartilage, leading to the adaptation of its classification to a ‘unique diarthrodial joint’ (DePalma, 1957; Dhawan et al., 2018; Jurik & Soerensen, 2007; Klein et al., 1995; Palastanga & Soames, 2018a; Tasnim et al., 2020). The presence of both or either hyaline cartilage and fibrocartilage at the joint surface may be attributed to two main factors. Previous research identified an infiltration of fibrocartilaginous fibers emanating from the attachment sites of the disc onto the joint surfaces, resulting in the development or appearance of fibrocartilage‐lined articular surfaces (Jurik & Soerensen, 2007). Additionally, other studies have emphasized a significant correlation between advancing age and the prevalence of fibrocartilage in these joint areas (DePalma, 1957; Jurik & Soerensen, 2007; van Tongel et al., 2012). While hyaline articular cartilage initially present on the joint surfaces is believed to undergo a transformation into fibrocartilage as age progresses, recent inquiries have cast doubt on these conclusions (Dhawan et al., 2018). Several investigations have identified instances of hyaline articular cartilage without any fibrocartilage on both joint surfaces, particularly on the clavicular surface of the adult SCJ (Dhawan et al., 2018; Epperson & Varacallo, 2021; Jurik & Soerensen, 2007; Warth, Lee, & Millett, 2014). The appearance of fibrocartilage on the articulating surfaces of the SCJ may also be a result of degenerative changes and the breakdown of cartilaginous tissue (Jurik & Soerensen, 2007). Given the current state of knowledge, it is recommended that further research be conducted on the cartilaginous composition at the joint surface.

### 2.1 Intra‐articular disc

The SCJ is divided internally by an intra‐articular disc residing between the articular surfaces of the clavicle and manubrium sterni (Barbaix et al., 2000; Dhawan et al., 2018; Epperson & Varacallo, 2021; Ghasemi et al., 2007; Sick & Ring, 1976). This disc effectively partitions the joint cavity into two separate compartments, creating isolated spaces within the articular cavity of the joint structure (Figures 1 and 2) (Drake et al., 2019). The intra‐articular disc is considered to be a continuation of the intra‐articular ligament, a fibrous intrinsic stabilizer of the SCJ, arising from the 1st sternochondral junction, attaching to the articular cartilage of the medial end of the clavicle, surrounding joint capsule (Tytherleigh‐Strong et al., 2017a). It attaches to the medial end of the clavicle via a broad fibrous attachment posterosuperiorly and has partial attachment to the 1st costal cartilage and surrounding joint capsule anterosuperiorly (Takeshige et al., 1998).

Similar articular discs are present at the temporomandibular joint, ACJ and ulnocarpal articulation, with crescent‐shaped menisci also being present in the tibiofemoral joint (Barlattani Jr et al., 2019; Fox et al., 2012; Kauer, 1975).

While the complete function of the intra‐articular disc is not always fully understood, it is thought to act in a similar manner as the meniscus of the knee and therefore functions to improve joint congruence, enhance joint stability, dissipate load force during movements of the upper limb and aid with load transmission across the joint (DePalma, 1959; Kelly et al., 1990; Morejon et al., 2022; Takeshige et al., 1998). Specific to the SCJ, the intra‐articular disc facilitates rotational movements of the clavicle and prevents the medial displacement of the clavicle on the sternum (DePalma, 1959; Takeshige et al., 1998; Terry & Chopp, 2000).

The intra‐articular disc of the SCJ is composed of a mixture of dense connective and fibrous tissue, having a fibrocartilaginous structure similar to the menisci of the knee; however, there is no agreement on its precise composition (Emura et al., 2009; Geneser, 1986; Ham & Cormack, 1979). Previous research identifies the intra‐articular disc to be fibrocartilaginous in its entirety (Adams & Wigley, 2016; Geneser, 1986; Ham & Cormack, 1979), however, more recently it has been described as being composed of dense connective tissue on its sternal end, with fibrocartilaginous tissue presenting only at its clavicular end (Emura et al., 2009; Tilmann, 2003b). Emura, Arakawa et al (Emura et al., 2009). identified the clavicular portion of the disc to be fibrocartilaginous due to the presence of round chondrocytes, while fusiform fibroblasts were solely identified within the sternal side of the disc, indicating a dense connective tissue structure. Echoing this finding, Tilmann (2003) describes the articular disc as a dense connective tissue structure. Fibrocartilage is generated from the connective tissue in response to substantial compressive loading, thereby suggesting that the clavicular side of the disc functions to withstand compressive load from the clavicular surface (Benjamin & Ralphs, 1998; Emura et al., 2009).

## 3 NEUROVASCULATURE OF THE SCJ

The SCJ is innervated by branches of the medial supraclavicular nerve (C3, C4) and the nerve to subclavius (C5, C6) (Barbaix et al., 2000; Dhawan et al., 2018; Epperson & Varacallo, 2021; Ghasemi et al., 2007; Sick & Ring, 1976). Its blood supply originates from branches of the internal thoracic and suprascapular arteries, both of which are derived from the subclavian artery (Hanisch et al., 2006). Additionally, a contribution from the 1st posterior intercostal artery has been noted (Barbaix et al., 2000). The attachments to the clavicular articular cartilage and 1st costal cartilage are highly vascular, with several small blood vessels throughout (Barbaix et al., 2000). Macroscopic examination by Barbaix et al (2000). identified blood vessels in the central portion of the disc, as well as at the articular surfaces of the clavicle and manubrium sterni, both of which had previously been described as being lined by articular cartilage (Barbaix et al., 2000). Healthy articular cartilage is avascular; therefore, this observation suggests that either the articular surfaces are composed of fibrocartilage rather than hyaline cartilage (as suggested above), similar to the arrangement at the temporomandibular joint, or breakdown to the cartilaginous extracellular matrix has led to decreased vascular resistance with subsequent vascular invasion as a result of progressive osteoarthritis (Barbaix et al., 2000). Neurovascular invasion of osteoarthritic articular cartilage has been observed in many joints throughout the body and is considered an important factor in the progressive formation of osteophytes associated with osteoarthritic joints (Ashraf et al., 2011; Qian et al., 2021; Suri et al., 2007; Xiaoshi et al., 2021). Previous research on weight‐bearing diarthrodial joints identified an increase in angiogenesis and blood vessel count throughout the cartilaginous material at high chondropathy scores or end‐stage osteoarthritis of the knee and hip joints (Ashraf et al., 2011; Ashraf & Walsh, 2008; Pauli et al., 2011; Shabestari et al., 2018). The articular cartilage at the hip and knee joints is subject to high compressive loading, creating dense and compact cartilage increasing the resistance to vascular invasion (Ashraf et al., 2011; Domzalski et al., 2017). Angiogenesis in weight‐bearing cartilage typically only occurs at advanced stages of osteoarthritic degeneration (Shabestari et al., 2018). While the SCJ is susceptible to loading from the upper limb, it experiences significantly less compressive weight‐bearing stress compared to the hip or knee. Consequently, its cartilaginous resistance against vascular invasion is reduced and therefore may occur during the preliminary stages of osteoarthritic degeneration (Shabestari et al., 2018). Histological assessment of blood vessel count and density compared to osteoarthritic changes in surrounding structures (as previously conducted on the osteoarthritic knee joint) may therefore provide a comprehensive overview of the articular surfaces and intra‐articular disc vascularity of the SCJ (Ashraf et al., 2011). It may also aid in developing the knowledge and understanding of osteoarthritic disease progression specific to the SCJ.

## 4 BIOMECHANICS OF THE SCJ

**FIGURE 3:**
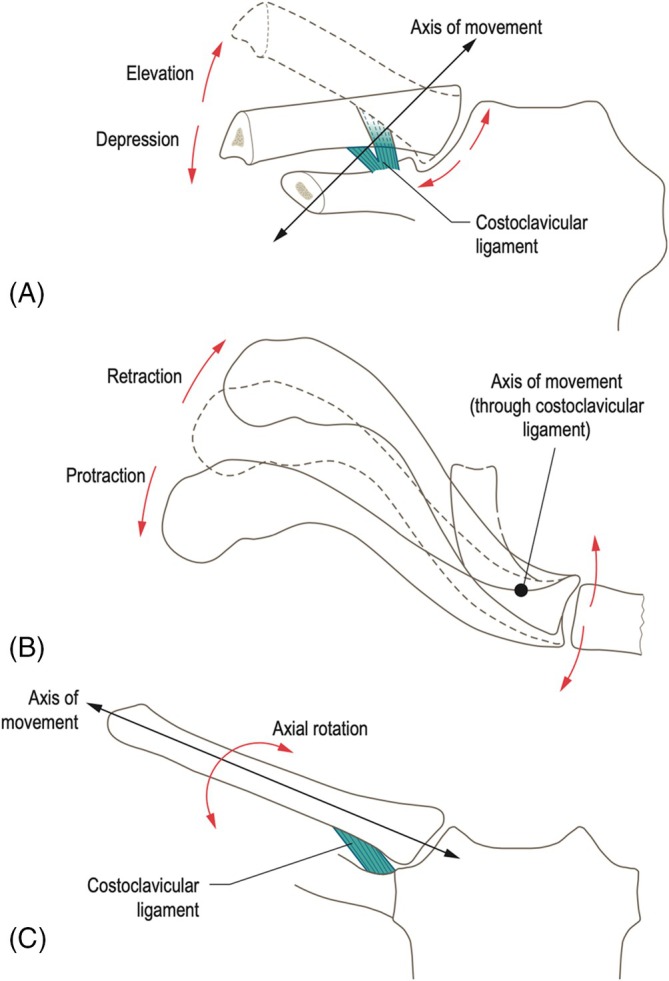
The sternoclavicular joint allows for movements of elevation and depression, retraction and protraction and axial rotation. Movements of (A) elevation/depression (Anterior view) and (B) retraction/protraction pivot around the costoclavicular ligament (Superior view). (C) Axial rotation of the clavicle passes through the center of the sternoclavicular and acromioclavicular joints (Anterior view) (Palastanga & Soames, 2012).

The medial end of the clavicle can vary in shape (circular, quadrangular, triangular, ovoid or prismatic) but typically presents as a bulbous saddle‐shaped structure with articular cartilage only present at its anteroinferior aspect (van Tongel et al., 2012; Walters et al., 2010; Warth, Lee, Campbell, & Millett, 2014). The posterosuperior aspect of the medial clavicle is void of articular cartilage and serves as an attachment point for the interclavicular ligament, sternoclavicular ligaments and the intra‐articular disc (Warth, Lee, Campbell, & Millett, 2014). The variably saddle‐shaped, medial end of the clavicle articulates indirectly with the smaller manubrium sterni via the fibrocartilaginous intra‐articular disc (van Tongel et al., 2012). As mentioned previously, this articulation is incongruent, mainly due to the variability in the size and shape of the medial end of the clavicle, leading to a lack of osseous stability (Palastanga & Soames, 2018a). While movements of the SCJ have been likened to a dynamic ‘ball and socket joint’ due to its capacity of movement, the SCJ is classified as a synovial double arthrodial joint. This dynamic joint exhibits a range of motions, allowing for a combined 60 degrees of elevation and depression (Palastanga & Soames, 2018a). These movements translate to approximately 10 cm of elevation and 3 cm of depression at the lateral end of the clavicle (Palastanga & Soames, 2018a). Furthermore, protraction and retraction movements maintain an approximate range of 35 °, resulting in 5 cm of protraction and 3 cm of retraction (Palastanga & Soames, 2018a). Additionally, the joint accommodates 20 to 40 ° of rotation around its longitudinal axis (Bontempo & Mazzocca, 2010; Dhawan et al., 2018; Epperson & Varacallo, 2021; Hobbs, 1968; Lowman, 1928; Palastanga & Soames, 2018a). Movements of elevation/depression and protraction/retraction occur through gliding movements between (i) the clavicle and the intra‐articular disc and (ii) between the intra‐articular disc and the manubrium of the sternum respectively (Dhawan et al., 2018). The rotational axis for movements of elevation and depression runs in a horizontal and oblique plane anterolaterally, while movements of protraction and retraction run in a vertical plane, obliquely and inferolaterally (Figure 3) (Palastanga & Soames, 2018a). With the exception of axial rotation, the pivotal point for these movements does not align with the center of the joint itself; instead, it occurs via the costoclavicular ligament (Figure 3) (Palastanga & Soames, 2018b). This subtle deviation from the central axis of motion at the joint center facilitates opposing movements at both the medial and lateral ends of the clavicle. (Palastanga & Soames, 2018b) Consequently, as the lateral end of the clavicle elevates, the medial end of the clavicle will concurrently depress, creating a dynamic counterbalance controlled and stabilized by the surrounding ligamentous structures (Figure 3) (Palastanga & Soames, 2018b).

Axial rotation of the SCJ is a passive movement, brought about by rotation of the scapula, transmitted to the SCJ through the costoclavicular ligament via the clavicle. Rotational movements occur as a result of three main factors: (i) incongruity of the articulating surfaces of the joint, (ii) the presence of the intra‐articular disc and (iii) distension of the ligamentous capsular thickenings (Palastanga & Soames, 2018a). Unlike other movements of the SCJ, the longitudinal axis for rotation extends through the center of the articular surface of the SCJ and neighboring ACJ (Figure 3C) (Palastanga & Soames, 2018a).

Movements of the SCJ contribute to abduction and flexion of the arm via elevation of the lateral end and rotation of the clavicle (Jurik, 2007). Any movement of the shoulder, which stimulates movement of the clavicle, leads to involvement of the SCJ (Palastanga & Soames, 2018a). Previous research confirms that the SCJ significantly influences the biomechanics of shoulder movement, adding a notable 4 ° of motion for every 10 ° of humeral elevation (Renfree & Wright, 2003). Minor forms of movement of the SCJ have also been identified during respiration (Jurik, 2007). These findings suggest that the SCJ remains under constant strain, rendering it vulnerable to degeneration over time (Jurik, 2007).

As previously highlighted, the SCJ is fundamentally unstable; however, stabilization is provided by surrounding soft tissue structures (Dhawan et al., 2018). Intrinsic static stabilization is provided by the intra‐articular disc, and intra‐articular and sternoclavicular ligaments, all of which aid to stabilize the forces crossing the joint from the upper limb to the axial skeleton, thereby decreasing the risk of displacement of the clavicle (Ghasemi et al., 2007).

The posterior sternoclavicular ligament forms a key structure in maintaining the horizontal stability of the SCJ while resisting anteroposterior translation (Warth, Lee, & Millett, 2014). Movements of elevation are limited by the costoclavicular ligament and subclavius muscle, while depression of the clavicle is restricted by the tension of the interclavicular ligament and articular disc (Palastanga & Soames, 2018a; Warth, Lee, & Millett, 2014). Anterior and posterior movements of the joint are limited by the anterior and posterior sternoclavicular and costoclavicular ligaments (Palastanga & Soames, 2018a; Warth, Lee, & Millett, 2014). Where soft tissue structures fail, movements of the SCJ (as a result of injury) are eventually limited by surrounding bony structures (Palastanga & Soames, 2018a).

Although the SCJ is not classified as a ‘weight‐bearing’ joint, it functions as a load‐bearing joint due to its exposure to diverse levels of distraction and compression from the upper limb (Sewell et al., 2013). The shear force of the joints of the upper limb is typically dismissed when compared to weight‐bearing joints, such as the knee or hip. The knee and hip joints experience substantial shear force loading due to surrounding and attaching musculature, as well as ground‐reaction and bony contact forces (Shelburne et al., 2004). These combined forces frequently lead to shear strains and calcification of the cartilaginous matrix initiating the onset of OA. (Shelburne et al., 2004) While the SCJ does not experience the same level of shear force that the knee and hip do, considerable loading has been identified from surrounding musculature and adjacent joints, particularly from the glenohumeral joint. Contact forces equivalent to 240% of the body weight have been previously identified at the glenohumeral joint of the shoulder when lifting a suitcase laterally (Klemt et al., 2018). Therefore, emphasizing the substantial mechanical load that may influence the upper extremity and emphasizing that these forces depend on the arm's position and the load borne by the upper limb throughout different movements (Sewell et al., 2013). Therefore, the medial portion of the SCJ is exposed to compressive forces from the medial clavicle, while the lateral part of the SCJ resists displacement in tension at the manubrium of the sternum (Sewell et al., 2013). Similar to how the meniscus of the knee helps to dissipate load force, the intra‐articular disc of the SCJ functions to transmit load forces.

Movements of the neighboring ACJ, particularly in the sagittal plane, are generally acknowledged to be more pronounced than those of the SCJ. However, rotational movements, in contrast, have been identified as significantly higher at the SCJ when compared to the ACJ (Irsay et al., 2020; Itoi et al., 2009; Palastanga & Soames, 2018a; Warth, Lee, & Millett, 2014). The ACJ functions to provide an increased range of movement at the pectoral girdle once maximal movement of the SCJ has been reached (Palastanga & Soames, 2018a).

The ACJ is similarly surrounded by a supporting joint capsule, stabilized by a series of adjacent ligaments and separating its joint space is a fibrocartilaginous disc of varying size and shape (Bontempo & Mazzocca, 2010). Although the ACJ and SCJ exhibit structural resemblances, the ACJ has been more prominently associated with degenerative changes (Renfree & Wright, 2003). This may be due to a number of factors, which include its anatomical location, joint articulations and disc degeneration (Mall et al., 2013). As previously stated, it is worth noting that the ACJ is situated laterally to the SCJ and is closer to the glenohumeral joint. Due to this external positioning, the ACJ is more susceptible to impact injuries (Kiel, Taqi, et al., 2022). Injury or damage to the ACJ or its surrounding supporting structures increases the likelihood of degeneration to the joint features, including degeneration to the intra‐articular disc (Kiel, Taqi, et al., 2022). Further to this, age related degeneration of the intra‐articular disc of the ACJ has been identified to occur, with reports of the disc being functionally insufficient by 40 years of age (Mall et al., 2013). Early degeneration of the disc and incongruent joint surfaces results in increased load force and heightened potential for cartilaginous degeneration (Mall et al., 2013).

## 5 DEGENERATION OF THE SCJ

Osteoarthritis is a broad term covering a group of joint diseases involving the articular cartilage, underlying bone, synovium and surrounding fibrocartilaginous and soft tissue structures (Pritzker et al., 2006). These conditions involve intricate microanatomical changes, including the degeneration of cartilage, followed by repair, chondrocyte death, and subsequent replication and proliferation (Pritzker et al., 2006). These changes typically lead to pain, friction and irritation as a result of increased degeneration of the articular cartilage, bone and surrounding structures (Morehead & Sack, 2003). It is important to recognize that pain may not consistently align with degenerative changes in osteoarthritis, attributed to the avascular and aneural nature of cartilaginous tissue (Hunter et al., 2013; Sofat et al., 2011). The onset of pain, therefore is usually due to the involvement of other joint structures such as bone and soft tissue (Sofat et al., 2011). Another hallmark feature of pain and joint sensitivity in OA is the thickening of the synovial membrane (Wang et al., 2018). This thickened membrane leads to an elevated production of synovial fluid, consequently raising the pressure within the joint, and increasing symptoms of pain (Wang et al., 2018).

Osteoarthritic degeneration of the SCJ can present as symptomatic or asymptomatic. Primary asymptomatic osteoarthritic degeneration of the SCJ impacts more than 90% of individuals above the age of 60 (Emura et al., 2007; Emura et al., 2009; Epperson & Varacallo, 2021; Ghasemi et al., 2007; Kier et al., 1986; Yood & Goldenberg, 1980). Symptomatic osteoarthritis of the SCJ is less commonly reported, with a prevalence of 53% in individuals over the age of 60 years, with initial indications of degeneration manifesting as early as 40 years of age (Kier et al., 1986; Thongngarm & McMurray, 2000). Most commonly, patients complain of pain and tenderness during movement of the shoulder joint, particularly during elevation (Sternheim et al., 2014).

Due to its frequency of use, the load‐bearing SCJ becomes progressively susceptible to osteoarthritic degeneration (Emura et al., 2009). Within weight‐bearing joints (e.g., knee, hip) the main factor leading to osteoarthritic degeneration appears to be continued repetitive high‐impact loading (Vincent et al., 2012). However, other factors such as injury, sternoclavicular compression contact time and sheer force within the joint generate frictional stresses and tension, which may lead to osteoarthritic joint degeneration over time (Vincent et al., 2012).

### 5.1 Diagnosis

Typically, osteoarthritis of the SCJ is characterized by degeneration of the articular surfaces of the joint and subchondral bone, with diagnostic criteria specifically focusing only on the integrity of these structures (Thongngarm & McMurray, 2000). A diagnosis of SCJ osteoarthritis begins with a thorough medical history assessment to eliminate potential injury or indications of referred pain (Tasnim et al., 2020). A common symptom of SCJ osteoarthritis is chest pain emanating from the joint: (Thongngarm & McMurray, 2000) pain and tenderness are often the initial diagnostic indicators of SCJ osteoarthritis (Thongngarm & McMurray, 2000). However, pain at the SCJ can often be misdiagnosed or confused with that emanating from other articular structures (e.g., axial skeleton, neighboring shoulder joint, costochondral articulations) reducing diagnostic accuracy (Yood & Goldenberg, 1980). Furthermore, arthritic pain radiating from the SCJ can often mimic the pain of ‘visceral origin’, leading to misdiagnosed chest pain involving cardiac and/or pulmonary complications (e.g., angina, pericarditis, myocardial infarction, pleurisy, pulmonary embolism) (Yood & Goldenberg, 1980). Subsequent steps involve joint imaging techniques, with the gold standard for osteophyte formation, cystic changes and sclerosis being computed tomography (CT) scanning (Dhawan et al., 2018; Lawrence & East, 2017). Due to the overlay of the clavicle, sternum and 1st rib, radiographs of the SCJ may be difficult to interpret (Dhawan et al., 2018; Logan et al., 2018). Similarly, the assessment of joint space narrowing is prone to inaccuracies as a result of overlapping structures, and consequently, it is not advisable for diagnosing SCJ osteoarthritis (Baker et al., 1988).

Establishing a definitive diagnosis of SCJ osteoarthritis often requires a considerable duration, given that its symptoms closely resemble those of other medical conditions (Jurik & Soerensen, 2007). The delayed confirmation of a diagnosis contributes to the advancement of untreated osteoarthritic disease progression (Jurik & Soerensen, 2007). Additionally current diagnostic criteria based on macroscopic inspection of the articular cartilage, coupled with symptoms of tenderness/pain, exhibit limited specificity, and overlook the comprehensive assessment of the entire joint structure. Degeneration and microanatomical changes of the intra‐articular disc are not considered clinically when forming a diagnosis of SCJ osteoarthritis.

### 5.2 Association between microscopic and macroscopic osteoarthritic degeneration

While many studies explore either the macroscopic or microscopic structure of the osteoarthritic joint, few studies explore both together. It is important to understand the link between macro and microscopic changes to develop a full picture of the current disease progression.

Previous research on various joints throughout the body has identified significant microscopic changes to macroscopically ‘healthy’ tissue. A combination of chondrocyte clustering, tidemark irregularities and derangement of articular collagen (each recognized as indicators of OA) have been observed to occur prior to any visible macroscopic signs of degeneration (Wilusz et al., 2013). The microenvironment of cartilaginous tissue therefore undergoes significant changes during the initial phases of OA, which gives rise to the observable macroscopic changes essential for clinical diagnosis. Initial microscopic changes typically surround the secretion of enzymes, such as matrix metalloproteinases or aggrecans from chondrocytes, which leads to the failure of maintained homeostasis between the synthesis and degradation of extracellular matrix (EM) components (Man & Mologhianu, 2014). This alteration in EM homeostasis results in increased water absorption, reduced proteoglycan content, and the hindrance of type II collagen production, leading to the degradation of existing collagen (Man & Mologhianu, 2014). Concurrently, there is a noticeable increase in chondrocyte apoptosis (Man & Mologhianu, 2014). Overtime, these microscopic changes lead to notable macroscopic degeneration, which is used to clinically diagnose OA. However, at this stage, significant irreversible microscope degeneration has already occurred. Therefore, early screening of both fibrocartilaginous and cartilaginous microscopic changes is essential. Ultrasound has previously been recommended for the assessment of early‐stage OA due to its capability of identifying microscopic changes to both fibrocartilage and cartilaginous structures (Huang et al., 2020; Nishitani et al., 2014). The importance of this diagnostic capability is further highlighted in the ‘investigations’ section.

Accurate identification and grading of both micro‐ and macroscopic osteoarthritic changes within a joint enables precise mapping of the relationship between these changes. This detailed mapping provides valuable pathological insights into the progression of osteoarthritic disease, enabling a better understanding of how alterations at the cellular and tissue levels influence overall joint anatomy and function. The microscopic and macroscopic changes to all components of the SCJ have yet to be assessed and should be considered for future research.

### 5.3 Intra‐articular disc and osteoarthritic degeneration

Previous studies have classified the intra‐articular disc of the SCJ as being one of three types: (i) fully intact (discoid type), (ii) only present peripherally (annular type) or (iii) lacking a posterior section (meniscus type) (de Palma, 1951; Emura et al., 2009; van Tongel et al., 2012). It is not known whether these types of intra‐articular discs are normal anatomical variants that occur during development or if they are the result of degeneration of the fibrocartilaginous interface in response to osteoarthritis (Emura et al., 2009). Previous studies surrounding the intra‐articular disc of the SCJ, have assumed that incomplete intra‐articular discs occur as a result of natural use or compression over time, as they tend to be more prevalent in the elderly, as well as occurring in combination with degenerative changes in neighboring articular structures; however, this has yet to be proven and remains an unconfirmed hypothesis (Barbaix et al., 2000; Kier et al., 1986; van Tongel et al., 2012). A sublabral recess, a common anatomical variant of the glenoid labrum, has been observed to be more prevalent in an older population group (De Coninck et al., 2016). However, the sublabral recess is not a result of pathological degeneration, but rather a morphological deviation occurring over time (De Coninck et al., 2016). Therefore, the identification of incomplete fibrocartilaginous discs within the aging population should not be automatically interpreted as a definitive sign of degenerative damage to the intra‐articular disc. Previous research by Kier, Wain et al. (Kier et al., 1986) suggested that incomplete articular discs of the SCJ are secondarily caused by osteoarthritic degeneration. However, in contrast, Emura, Matsuzaki et al. (Emura et al., 2007) identified no microscopic signs of osteoarthritic degeneration (micro tears, myxoid degeneration or chondrocyte cloning) within incomplete discs (meniscoid and ring type). Incomplete disc types have also been identified to be associated with subluxation or dislocation injuries of the SCJ (Tytherleigh‐Strong et al., 2017a).

### 5.4 Fibrocartilaginous and cartilaginous osteoarthritic disease progression

Previous research has identified significant microanatomical changes to both the intra‐articular disc and hyaline cartilage of osteoarthritic SCJs (Emura et al., 2007; Ghasemi et al., 2007). These changes affect the cellularity, collagen alignment, surface fibrillation, cyst formation and glycosaminoglycan (proteoglycan) content within the osteoarthritic SCJ (Emura et al., 2007; Ghasemi et al., 2007). Chondrocyte cloning has also been identified within the cartilaginous tissue, which shows the initiation of attempted repair, a key trait of osteoarthritic disease progression (Ghasemi et al., 2007; Park et al., 2021). Within the intra‐articular disc, myoxid degeneration, chondrocyte clustering and fiber delamination have been identified in response to osteoarthritis, leading to weakening tissue structures (Emura et al., 2007). Interestingly, these osteoarthritic changes were more apparent in the intra‐articular disc as opposed to the osteoarthritic changes in the articular cartilage (Ghasemi et al., 2007). This provides additional indications of the potential involvement of the intra‐articular disc in the initial phases of osteoarthritic degeneration. However, while changes in the osteoarthritic SCJ were indeed observed, they were not subjected to grading, and the correlation between grades of osteoarthritic degeneration in the disc and cartilage was not investigated (Emura et al., 2007). Grading of the osteoarthritic micro‐changes of the disc and articular surfaces is vitally important as it develops the understanding behind the disease progression of SCJ OA. Therefore, a thorough understanding of the initial stages of the osteoarthritic disease progression at the SCJ remains unknown.

Although the impact of osteoarthritic disease progression on the fibrocartilaginous disc of the SCJ is not yet fully understood, prior research on the hip and knee has revealed comparable changes in both cartilage and fibrocartilaginous structures. These changes include variations in cellularity, surface integrity, collagen alignment, and proteoglycan content, mirroring the osteoarthritic conditions observed in the SCJ (Ashraf et al., 2011; Domzalski et al., 2017; Pauli et al., 2011; Sun et al., 2012). Osteoarthritic changes identified within the articular surfaces and fibrocartilaginous structures of the knee and hip were graded (Dyment et al., 2015; Pauli et al., 2011; Sun et al., 2012). Grading scales such as those used by Sun, Mauerhan et al. (Sun et al., 2010), Domzalski, Synder et al. (Domzalski et al., 2017) and Pauli, Grogan et al. (Pauli et al., 2011) at the knee have been developed and validated through a series of robust sampling of both healthy and osteoarthritic menisci/labrum/cartilage with excellent inter‐reader agreement and reliability (Pauli et al., 2011). Grading osteoarthritic changes within surrounding tissues allows for a better understanding of the disease progression and involvement of the affected joint. Consequently, considering this approach is advisable for use within the SCJ. Once grades of both joint structures were determined, the relationship between graded changes of the osteoarthritic cartilage and fibrocartilage of the knee and hip were assessed and a significant correlation identified (Ashraf et al., 2011; Domzalski et al., 2017; Pauli et al., 2011). Therefore, indicating that increased articular cartilage degeneration corresponds to a parallel increase in fibrocartilage degeneration, and conversely.

The active participation of fibrocartilaginous structures within an osteoarthritic joint raises the prospect that these structures may play a significant role in initiating joint OA (Dyment et al., 2015; Petersson, 1983). Supporting this notion, Dyment, Hagiwara et al. (Dyment et al., 2015) highlighted significant degeneration and remodeling of the meniscus' fibrocartilage, while no noticeable histological alterations were observed in the articular cartilage covering the joint surfaces (Dyment et al., 2015). Following injury to the knee, degeneration to the fibrocartilage began as early as 2 weeks from the date of injury with progression to severe deterioration and osteophyte formation within 4 weeks, therefore, highlighting the direct role of fibrocartilage within osteoarthritic disease progression (Dyment et al., 2015).

While these findings are specific to osteoarthritic weight‐bearing joints, similar results have been identified within load‐bearing osteoarthritic joints of the upper limb. A previous study on the ACJ identified that degenerative changes to the fibrocartilaginous intra‐articular disc precede osteoarthritic degeneration to surrounding cartilage (Petersson, 1983). This study used a robust sample size of 85 cadavers (resulting in 168 ACJs) with a mean age of 69 years and a relatively equal representation between genders (Petersson, 1983). Degenerative changes are significantly correlated with age, highlighting the increase in osteoarthritic degenerative changes within an aging population (Petersson, 1983). Grading of cartilaginous degeneration of the osteoarthritic ACJ has been conducted with grades of degeneration ranging from I‐III, where grade I indicates superficial degeneration and Grade III involves full‐thickness cartilaginous degeneration (Petersson, 1983). The grading system used within this study did not consider the degenerative changes to the intra‐articular disc, instead changes to the disc were observed separately. Within the osteoarthritic ACJ, Petersson (1983) identified severe to maximal degeneration to the intra‐articular disc, when cartilage degeneration scores ranged between grades II or III (Petersson, 1983). Notably, the extent of degeneration identified within the fibrocartilaginous discs exceeds degeneration even at the highest grades observed in the articular cartilage. This suggests that the degeneration process begins within the fibrocartilaginous disc before progressing to affect the articular cartilage (Petersson, 1983).

These findings emphasize the importance of assessing fibrocartilaginous structures when evaluating early‐stage osteoarthritic degeneration. It suggests that when an initial diagnosis of SCJ osteoarthritis is made, significant degeneration of the intra‐articular disc may already have occurred. The SCJ and ACJ share a similar anatomical structure and triplanar movements and therefore the disease progression identified within the ACJ may similarly be present at the SCJ. However, as mentioned previously, the ACJ is typically involved in higher rates of injury and degeneration, therefore, further investigation into the osteoarthritic disease progression of the SCJ is necessary.

### 5.5 Investigations and current management

The majority of drug, genetic and management therapies focus on the treatment of joint osteoarthritis following the onset of articular cartilage destruction; however, initiation of these treatment regimens following the onset of fibrocartilaginous changes could result in higher efficacy and improved patient outcomes (Dyment et al., 2015). The early identification of changes to the intra‐articular disc of the osteoarthritic SCJ may aid in delaying or inhibiting the progression of painful joint destruction (Dyment et al., 2015). As previously mentioned, the gold standard for SCJ OA diagnosis is through the use of a CT scan to assess bony and soft tissue changes within the joint (Dhawan et al., 2018; Lawrence & East, 2017). However, the presence of these changes typically indicate advanced osteoarthritic degeneration as a result of extensive bony involvement coupled with significant cartilaginous degeneration. Previous research on the lesser metatarsophalangeal joints of the foot identified that degenerative changes to the fibrocartilaginous plantar plate can be assessed clinically by ultrasound and MRI (Linklater & Bird, 2016). Much like the intra‐articular disc of the SCJ, the plantar plate is a synovial joint‐stabilizing fibrocartilaginous structure (Hatch, 2022). However, in contrast to the SCJ, the plantar plate is positioned at the base of the metatarsophalangeal joint rather than within the joint itself (Hatch, 2022). The entirety of the plantar plate has been successfully assessed using MRI and ultrasound, despite its deep location and overlying musculature. These findings highlight the potential efficacy of employing MRI and/or ultrasound in investigating the intra‐articular disc of the SCJ. This may be especially helpful in the early diagnosis of individuals with a family history of osteoarthritis and/or previous shoulder injuries, as a diagnosis may be made before the initiation of deformity and pain symptoms. The use of MRI in identifying degenerative fibrocartilaginous changes within a joint is highly effective, due to its sensitivity in visualizing the soft tissue structures of a joint. Previous literature has identified a high degree of MRI specificity in identifying the fibrocartilaginous meniscus of the knee and the triangular fibrocartilage of the wrist, with a diagnostic sensitivity of 88% and specificity of 94% (Lefevre et al., 2016; Mackenzie et al., 1996). This is further echoed on the examination of the ACJ through MRI (Heers et al., 2007). Within this study, the ACJs from a mixture of cadaveric specimens as well as volunteers with no history of shoulder injury were assessed (Heers et al., 2007). All aspects of the joint structure, including the disc were identified, with signs of degeneration identified, thereby highlighting the effectiveness of assessing the integrity of the joint structure as a whole using MRI (Heers et al., 2007). All aspects of the fibrocartilaginous intra‐articular disc can be clearly identified and examined through routine MRI, rendering it an optimal tool to assess degenerative changes to the SCJ (Aslam et al., 2002; Ernberg & Potter, 2003; Olivier et al., 2022). However, MRI for routine examinations is costly and does not allow for real‐time imaging (Lento & Primack, 2008).

Dynamic imaging of the SCJ throughout movements of the shoulder allows further visualization of the disc in order to assess for (i) disc type and (ii) regions of degeneration, if present. High‐frequency ultrasound has been identified to be an effective alternative to MRI in the full visualization of the SCJ (Olivier et al., 2022). Low cost, real‐time dynamic imaging and increased availability allow ultrasound to be an excellent alternative to MRI in the examination of the SCJ (Olivier et al., 2022). Further to this, as previously mentioned, previous research has revealed that ultrasound exhibits high sensitivity and capability to discern microscopic osteoarthritic changes within cartilaginous structures before they manifest as macroscopic degeneration (Huang et al., 2020; Nishitani et al., 2014; Wang et al., 2016). This underscores its utility in diagnosing early‐stage osteoarthritis, therefore highlighting it as an effective tool in assessing micro‐changes of the joint structures of the SCJ.

A recent study by Olivier, Kasprzak et al. (Olivier et al., 2022) stated that high‐frequency ultrasound is sufficient in viewing the intra‐articular disc of the SCJ with the articular disc being visible in 66% of the sample of 59 volunteer patients. However, in patients who are overweight or if excess intra‐articular gas is present, visualization is poor (Olivier et al., 2022). This is the first study of its kind to assess the efficacy of ultrasound in the assessment of the intra‐articular disc of the SCJ (Olivier et al., 2022). Previous research suggests that while fibrocartilaginous structures within a joint may not be clearly identified, signs of degeneration such as cyst formation, oedema, or fibrillation can be clearly identified through the use of ultrasound (Lento & Primack, 2008). This study utilizes a relatively small sample size (*n* = 59), with a low median age (35 years), and therefore may not offer a complete understanding of the complete SCJ (Olivier et al., 2022). It is essential to conduct further investigations to delve deeper into the efficacy of ultrasound sensitivity in detecting degenerative changes within the intra‐articular disc of the SCJ. To achieve a more comprehensive understanding, future research should encompass a broader range of participants to ensure the generalizability and robustness of the findings.

Precise imaging through ultrasound of the SCJ intra‐articular disc and surrounding structures may aid in enhancing the knowledge and understanding of osteoarthritic disease progression and the microanatomical changes, which occur in response to pathological arthropathies. Highlighting these changes and developing a better understanding of the morphology of the intra‐articular disc may allow for the early introduction of medical intervention in response to early signs of degeneration and thereby improve treatment outcomes in response to SCJ osteoarthritis.

Treatment modalities for the osteoarthritic SCJ generally involve surgical repair. Open resection arthroplasty of the SCJ is typically recommended at advanced stages of joint dysfunction as a result of symptomatic osteoarthritic degeneration (Warth, Lee, Campbell, & Millett, 2014). This procedure involves the removal of the arthritic joint surfaces to reduce the degree of joint friction, inflammation and pain (Tytherleigh‐Strong & Van Rensburg, 2017; Warth, Lee, Campbell, & Millett, 2014). While this procedure has had positive outcomes and good patient satisfaction, it is important to acknowledge that this procedure comes with inherent risks and potential complications (Warth, Lee, Campbell, & Millett, 2014). One primary concern of an open resection at this level is the proximity of vital mediastinal structures (Tytherleigh‐Strong et al., 2017a; Tytherleigh‐Strong & Van Rensburg, 2017). Any inadvertent damage to these structures can lead to serious and potentially fatal complications.

An arthroscopic decompression of the SCJ is therefore a preferred alternative as it achieves similar results as the open procedure but reduces some of the associated risks (Tytherleigh‐Strong et al., 2017a; Tytherleigh‐Strong & Van Rensburg, 2017; Warth, Lee, Campbell, & Millett, 2014). While there is still a risk of possible infection post‐operatively, there is only a minimal risk to surrounding mediastinal structures (Tytherleigh‐Strong et al., 2017a; Tytherleigh‐Strong & Van Rensburg, 2017). A previous case study surrounding arthroscopic decompression of the SCJ identified a tear to the intra‐articular disc, which accompanied chondral degeneration to both joint surfaces and synovitis (Warth, Lee, Campbell, & Millett, 2014). Interestingly, the intra‐articular disc tear was only observed in conjunction with chondral degeneration on both joint surfaces and was absent in cases with isolated degeneration of the clavicle facet (Warth, Lee, Campbell, & Millett, 2014). This association between intra‐articular disc tears and chondral degeneration has been corroborated by other studies (Lawrence & East, 2017; Tytherleigh‐Strong et al., 2017a; Tytherleigh‐Strong et al., 2017b). Therefore, ultrasound examination of the tissue may aid in highlighting areas of degeneration of the disc prior to tearing.

## 6 CONCLUSION

The changes in the intra‐articular disc and hyaline cartilage of osteoarthritic SC joints have been evaluated based on various factors, such as cell count, collagen alignment, surface fibrillation, cyst formation, and glycosaminoglycan (proteoglycan) content. The evaluation has revealed significant alterations in these factors, however, no grading or classification of the microanatomical changes in relation to the arthritic changes has been conducted. In the absence of an established grading system for the changes that occur in the fibrocartilaginous disc, there is uncertainty regarding the relationship between its degeneration and that of the articular cartilages. Grading and mapping the micro−/macroscopic changes in both fibrocartilaginous and cartilaginous tissue will aid in enhancing the therapeutic insights behind the disease progression of OA. Research on similar diarthrodial joints has shown that osteoarthritic degeneration of associated fibrocartilaginous structures precedes degeneration of the articular cartilage, acting as an early indicator of osteoarthritis. This review highlights the need for further investigation into the relationship between microanatomical changes to all components of the osteoarthritic SCJ, specifically its fibrocartilaginous intra‐articular disc and the development of a thorough grading system.

Current recommendations of imaging modalities to view the intra‐articular disc include MRI and high‐frequency micro‐ultrasound imaging; however, ultrasound sensitivity to assess for early microscopic degeneration within the fibrocartilaginous disc needs to be assessed further. Understanding the relationship and progression of degeneration between the intra‐articular disc and joint articular surfaces will improve the understanding of osteoarthritic disease progression of the SCJ and promote its early diagnosis.
